# The ionic salts with super oxidizing ions O_2_^+^ and N_5_^+^: Potential candidates for high-energy oxidants

**DOI:** 10.3389/fchem.2022.1005816

**Published:** 2022-09-21

**Authors:** Xinbo Yang, Nan Li, Yuchuan Li, Siping Pang

**Affiliations:** ^1^ School of Material Science & Engineering, Beijing Institute of Technology, Beijing, China; ^2^ School of Mechatronical Engineering, Beijing Institute of Technology, Beijing, China

**Keywords:** super energetic oxidizer, O_2_^+^ and N_5_^+^ ionic salts, detonation performance, quantum chemical calculation, energetic materials

## Abstract

As an important component of energetic materials, high-energy oxidant is one of the key materials to improve their energy. The oxidizability of oxidant directly determines the intensity of combustion or explosion reaction. It is generally believed that when the nature of reductant is certain, the stronger the oxidizability, the more intense the reaction. Dioxygenyl cation (O_2_^+^) and pentazenium cation (N_5_^+^) are two kinds of super oxidizing ions, which oxidizability are comparable to that of fluorine. A series of high energetic ionic salts with O_2_^+^, N_5_^+^ and various anions as active components are designed, and the results show that: 1) Most ionic salts have appropriate thermodynamic stability, high density (up to 2.201 g/cm^3^), high enthalpy of formation (up to 1863.234 kJ/mol) and excellent detonation properties (up to 10.83 km/s, 45.9 GPa); 2) The detonation velocity value of O_2_ (nitrotetrazole-*N*-oxides) and O_2_B(N_3_)_4_ exceed 10.0 km/s, and the detonation pressure exceed 45.0 GPa because of the O_2_^+^ salts have higher crystal density (g/cm^3^) and oxygen balance than that of N_5_^+^salts; 3) With a higher nitrogen content than O_2_^+^, the N_5_^+^ salts have higher enthalpy of formation, which exceed 330 kJ/mol than that of O_2_^+^ salts; 4) The linear spatial structure of N_5_^+^ leads the salts to reduce their density. Encouragingly, this study proves that these super oxidizing ions have the potential to become high-energy oxidants, which could be a theoretical reference for the design of new high energetic materials.

## 1 Introduction

An oxidant is a reactant that oxidizes or removes electrons from other reactants during a redox reaction, which is widely used in aerospace propellants, explosives, and pyrotechnics (Connelly and Geiger, 1996) . The oxidants, which directly participate in combustion or explosion reactions, is one of the indispensable components for such chemical reactions. For example, dinitramide salts (Venkatachalam et al., 2004) and perchlorates (Zeng and Bernstein, 2019) are added to energetic formulas to increase the oxygen balance and density of the whole system.

In energetic materials, the functions of oxidants are: 1) Speeding up burning more intensely; 2) Causing materials that are normally not readily combustible in air to burn more readily; 3) Causing combustible materials to burn spontaneously without a source of ignition. It is generally agreed that the oxidant with strong oxidizability can significantly improve the energetic properties of mixed explosive when the nature of reductant is certain.

However, traditional oxidants (such as ammonium perchlorate, hydrazine perchlorate, nitroyl perchlorate, hydroxylamine perchlorate, etc.) often contain halogen elements, which are easy to cause harm to human body and the environment; The energy level of existing high-energy oxidants such as 1,3,5-triamino-2,4,6-trinitrobenzene (TATB), 1,1-diamino-2,2-dinitroethylene (FOX-7), dihydroxylammonium 5,5′-bistetrazole-1,1′-diolate (TKX-50), octahydro-1,3,5,7-tetranitro-1,3,5,7-tetrazocine (HMX), hexahydro-1,3,5-trinitro-1,3,5-triazine (RDX), and hexanitrohexaazaisowurtzitane (CL-20) has reached the limit of energetic oxidants. The design and development of energetic ionic salts with super oxidation and gas products are expected to promote the innovative development of traditional energetic materials. Excitingly, the O_2_^+^ and N_5_^+^ are two super oxidizing cation systems, and their oxidizing power are comparable to that of fluorine gas. These two cations consist only of oxygen and nitrogen elements, respectively, which ensures that their reaction products are likely to be nitrogen and various gaseous non-metallic oxides.

Dioxygenyl hexafluoroplatinate containing O_2_^+^ was first synthesized by Bartlett et al. (Bartlett and Lohmann, 1962) in 1962, which started the wave of research of dioxygenyl cation. Subsequently, various O_2_^+^ salts containing fluorine anions [such as O_2_^+^MF_6_^-^ (M = P, As, Sb, Bi, Pt, Ru, Rh, Pd, or Au), O_2_^+^M_2_F_11_^−^ (M = Sb, Bi, Nb, or Ta), Or O_2_^+^MF_4_^-^ (M = B)] were successively characterized (Grill et al., 1970; Nikitin and Rosolovskii, 1971; Goetschel and Loos, 1972; McKee and Bartlett, 1973; Christe et al., 1974; Edwards et al., 1974; Gillespie and Schrobilgen, 1974; DiSalvo et al., 1975; Griffiths et al., 1975; Holloway and Schrobilgen, 1975; Rigny and Falconer, 1975). In 1973, Stein et al. (1973) used O_2_SbF_6_ to remove xenon and radon from the atmosphere, and oxygen was released after the reaction. It directly proved that O_2_^+^ has strong oxidizability, so that it can directly oxidize the inert gas and release oxygen. In 1976, Falconer et al. (1976) synthesized O_2_^+^PdF_6_^−^, that is, Pd (V) can be stabilized to hexafluorides by forming a complex salt with dioxygenyl cation. In 1976, Christie et al. (1976) synthesized and characterized another new O_2_^+^ salt: O_2_^+^GeF_5_^−^. This substance was prepared by UV photolysis of GeF_4_-F_2_-O_2_ mixture in quartz at −78°C. In 1991, Fisher et al. (1991) studied the reaction between O_2_^+^ and CF_4_/C_2_F_6_, and found that FCO^+^ and F_2_CO^+^ would be generated after the reaction, which shows the super oxidizability of O_2_^+^. However, the above studies focus on the stability and oxidizability of dioxygenyl ions, and did not involve too much research on energetic materials. Holfter studied the reaction between O_2_^+^BF_4_^−^ and activated sodium azide in the presence of metallic aluminum in 1997 (Holfter et al., 1997). The reaction produces NaBF_4_, N_2_ and Al_2_O_3_, and releases 434 kcal/mol of heat, which fully shows that O_2_^+^BF_4_^−^ is a high energy density material.

In addition, the all-nitrogen cation N_5_^+^ is another attractive super oxidizing ion compared to the O_2_^+^. Christe et al. (1999) first reported the synthesis and characterization of N_5_^+^AsF_6_^−^ in 1999, which has a chain structure and could be a high energy density material. This study shows that N_5_^+^AsF_6_^−^ is a white solid slightly soluble in anhydrous HF, which is relatively stable at 22 ℃ , and can be stored for several weeks without decomposition at −78 ℃ . In 2001, Vij et al. (2001) prepared another N_5_^+^ salt: N_5_^+^Sb_2_F_11_^−^, and studied the oxidation of this salt. The results show that N_5_^+^ is a super strong single electron oxidant, which can oxidize NO, NO_2_ and Br_2_, but cannot oxidize Cl_2_, Xe and O_2_. In 2003, Wilson et al. (2003) prepared (N_5_^+^)_2_SnF_6_^2-^, N_5_^+^SnF_5_^−^, and N_5_^+^B(CF_3_)^-^ by combining N_5_^+^ and multi-electron anions. In 2004, Dixon et al. (2004) confirmed that both N_5_^+^N_3_^−^ and N_5_^+^N_5_^−^ were unstable structures by theoretical calculation, but pointed out that the stability prediction of individual ionic compounds could not represent the stability of such substances. In the same year, Haiges et al. (2004) also published the synthesis and characterization of N_5_^+^ high energy density materials, which include N_5_^+^[P(N_3_)_6_]^-^, N_5_^+^[B(N_3_)_4_]^-^, N_5_^+^[HF_2_]^-^·nHF, N_5_^+^[BF_4_]^-^, N_5_^+^[PF_6_]^-^, and N_5_^+^[SO_3_F]^-^.

**SCHEME 1:**
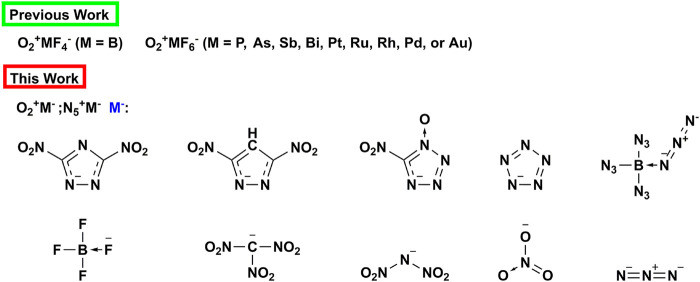
Fluorinated anions for stabilizing O_2_^+^ in previous works and energetic anions used in this paper to stabilize O_2_^+^ and N_5_^+^.

Based on the previous researches, the super oxidation and potential energetic properties of N_5_^+^ and O_2_^+^ greatly encourage us to further study the application potential of these substances in the field of energetic materials. In this paper, N_5_^+^ and O_2_^+^ are used as the cationic components of energetic ionic salts, while the polyazole rings, BF_4_^−^, B(N_3_)_4_^-^, NO_3_^−^, C(NO_2_)_3_^-^, and N_3_^−^are used as anions to design two new types of energetic ionic salts (see Scheme 1). By means of quantum chemical calculation, we studied the physical properties (density, enthalpy of formation), stability and detonation properties of these two energetic ionic salts. We hope that this research work can deepen people’s understanding of super oxidizing ions, and provide some reference and theoretical support for the development of new energetic oxidants.

## 2 Calculation method

The geometries of each structure corresponding to the stationary point on the potential energy surface (PES) of the specie studied were fully optimized using density functional theory at the M06-2X/6-311+G (d, p) level with Gaussian 09 (Wong et al., 2002; Zhao and Truhlar, 2007; Frisch et al., 2016). Zero-point energies and thermal corrections to enthalpy (the correction factor is 0.97) and Gibbs free energies are computed at the same DFT level. Single-point electronic energies are afterward refined using PWPB95 (Goerigk and Grimme, 2011) functional in conjunction with a def2-QZVP (Weigend and Ahlrichs, 2005) by ORCA (Neese, 2011). The molecular van der Waals surface electrostatic potential distribution of anions and cations were obtained with Multiwfn (Lu and Chen, 2012a; b).

The crystal density (*ρ*) was estimated using the improved equation (Rice and Byrd, 2013) shown as follows Eq. 1.

$$\rho =\alpha \frac{M}{V_{m}}+\beta \left(\frac{{\bar{V}}_{S}^{+}}{A_{S}^{+}}\right)+\gamma \left(\frac{{\bar{V}}_{S}^{-}}{A_{S}^{-}}\right)+\delta$$ (1)

where *M* is the molecular mass of the compound. *V*_*m*_ is the volume of the isolated gas molecule. The $A_{S}^{+}$ (Bohr^2^) is the portion of a cation’s surface which has a positive electrostatic potential, and the ${\bar{V}}_{S}^{+}$ (kcal/mol) is the average value of positive electrostatic potential; the $A_{S}^{-}$ and ${\bar{V}}_{S}^{-}$ are the analogous quantities for an anion. These four parameters are calculated by Multiwfn (Lu and Chen, 2012a).

The volume (*V*) of ionic compounds (*M*_*p*_ *X*_*q*_) (Jenkins et al., 1999; Rice et al., 2007) is estimated using the sum of the respective volumes of cations and anions by Eq. 2.

$$V=pV_{M^{+}}+qV_{X^{-}}$$ (2)

where $V_{M^{+}}$ and $V_{X^{-}}$ are the volume of the cation *M* ^+^ and anion *X* ^−^, respectively. The *p* and *q* are the number of cation *M* ^+^ and anion *X* ^−^ per formula unit, respectively.

**SCHEME 2:**
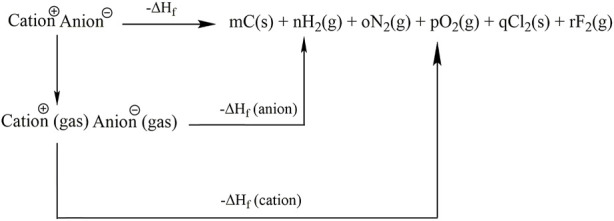
Born-Haber energy cycle for the enthalpy of formation of energetic salts.

As shown in Scheme 2, based on the Born-Haber energy cycle, the enthalpy of formation (Gao et al., 2007) of ionic compounds can be predicted by Eq. 3:

$$\Delta H_{f}^{0}\left(salt,\;298\;K\right)=\Delta H_{f}^{0}\left(cation,\;298\;K\right)+\Delta H_{f}^{0}\left(anion,\;298\;K\right)-\Delta H_{L}$$ (3)

where $\Delta H_{L}$ is the lattice energy of the salts which can be calculated by Eq. 4 put forward by Jenkins et al. (2002).

$$\Delta H_{L}=U_{POT\;}+\left[p\left(n_{M}/2-2\right)+q\left(n_{X}/2-2\right)\right]RT$$ (4)

where $n_{M}$ and $n_{X}$ rely on the nature of the ions M_p+_ and X_q-_, respectively, are equal to 3 when they are monatomic ions, 5 when they are linear polyatomic ions, and 6 when they are nonlinear polyatomic ions. In addition, the lattice potential energy ( $U_{POT}$ ) can be estimated by Eq. 5:

$$U_{POT}=\gamma {\left(\rho /M\right)}^{1/3}+\delta$$ (5)

where ρ (g cm^−3^) is the density and *M* (g mol^−1^) is the chemical formula mass of the ionic salt. For 1:1 (charge ratio) salts, the fitted coefficients γ and δ are 1981.2 kJ mol^−1^·cm and 103.8 kJ mol^−1^; for 1:2 salts, they are 8,375.6 kJ mol^−1^·cm and -178.8 kJ mol^−1^; for 2:2, they are 6,864.0 kJ mol^−1^·cm and 732.0 kJ mol^−1^.

The detonation velocity and pressure were predicted by empirical Kamlet−Jacobs in EXPLO5 (v6.05) program (Sućeska, 2018) by Eqs 6, 7:

$$D=1.01{\left(N{\bar{M}}^{\frac{1}{2}}Q^{\frac{1}{2}}\right)}^{\frac{1}{2}}\left(1+1.30\rho \right)$$ (6)

$$P=1.558\rho^{2}N{\bar{M}}^{\frac{1}{2}}Q^{\frac{1}{2}}$$ (7)

where *D* is the detonation velocity (km/s), *P* is the detonation pressure (GPa), *N* is the moles of detonation gases per gram explosive, *M̅* is the average molecular weight of these gases, *Q* is the heat of detonation (cal/g), and *ρ* is the loaded density of explosives (g/cm^3^) and is replaced by the theoretical density here.

**SCHEME 3:**
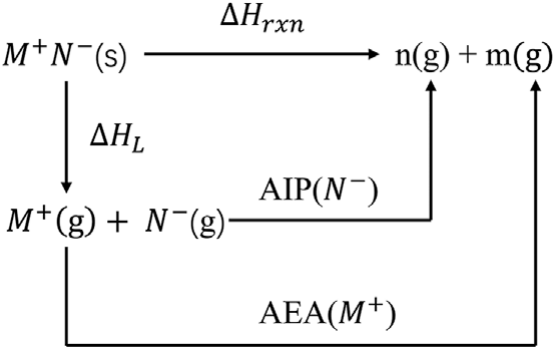
Born-Haber energy cycle for the reaction enthalpy change of energetic salts.

For ionic compounds (*M*_*p*_ *X*_*q*_), the Gibbs free energy change of formation ( $∆G_{rxn}\left(salt\right)$ , Eq. 8) can be used to describe whether it can be decomposed in thermodynamics. The reaction enthalpy change ( $∆H_{rxn}\left(salt\right)$ , Eq. 9) of ionic salts can be obtained based on the Born-Haber cycle, (see Scheme 3), relying on the lattice energy ( $∆H_{L}$ ) of the ionic salt, the adiabatic electron affinity ( $AEA\left(M^{+}\right)$ ) of the cation and the adiabatic ionization potential ( $AIP\left(X^{-}\right)$ ) of the anion (Dixon et al., 2004). For entropies of the title salts ( ${∆S}_{rxn}\left(salt\right)$ , Eqs 10, 11) could be predicted by employing relationships which were developed by Glasser and Jenkins (Glasser and Jenkins, 2004) for organic solids.

$$∆G_{rxn}\left(salt\right)=∆H_{rxn}\left(salt\right)-T∆S_{rxn}\left(salt\right)$$ (8)

$$∆H_{rxn}\left(salt\right)=∆H_{L}+IP\left(X^{-}\right)+EA\left(M^{+}\right)$$ (9)

$$∆S_{rxn}\left(salt\right)=S_{salt}-S_{M^{+}}-S_{X^{-}}$$ (10)

$$S_{salt}=1.258\left(\frac{M}{\rho }\right)+57$$ (11)

## 3 Results and discussion

### 3.1 Configuration

**FIGURE 1:**
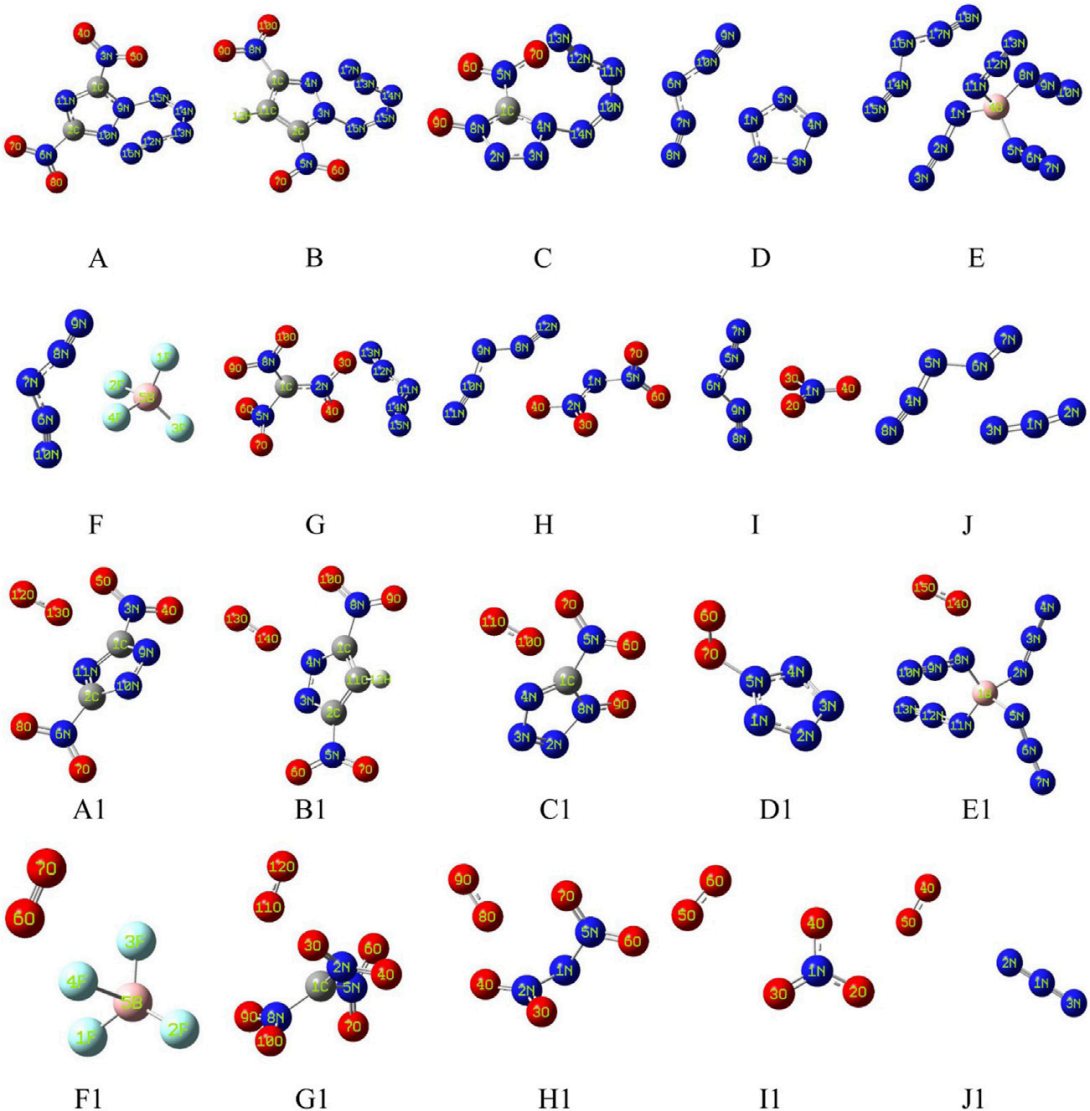
Theoretically designed energetic compounds containing N_5_^+^ **(A–J)** and O_2_^+^ **(A1–J1)** ions at M06-2X/6-311+G (d, p) theory.

**FIGURE 2:**
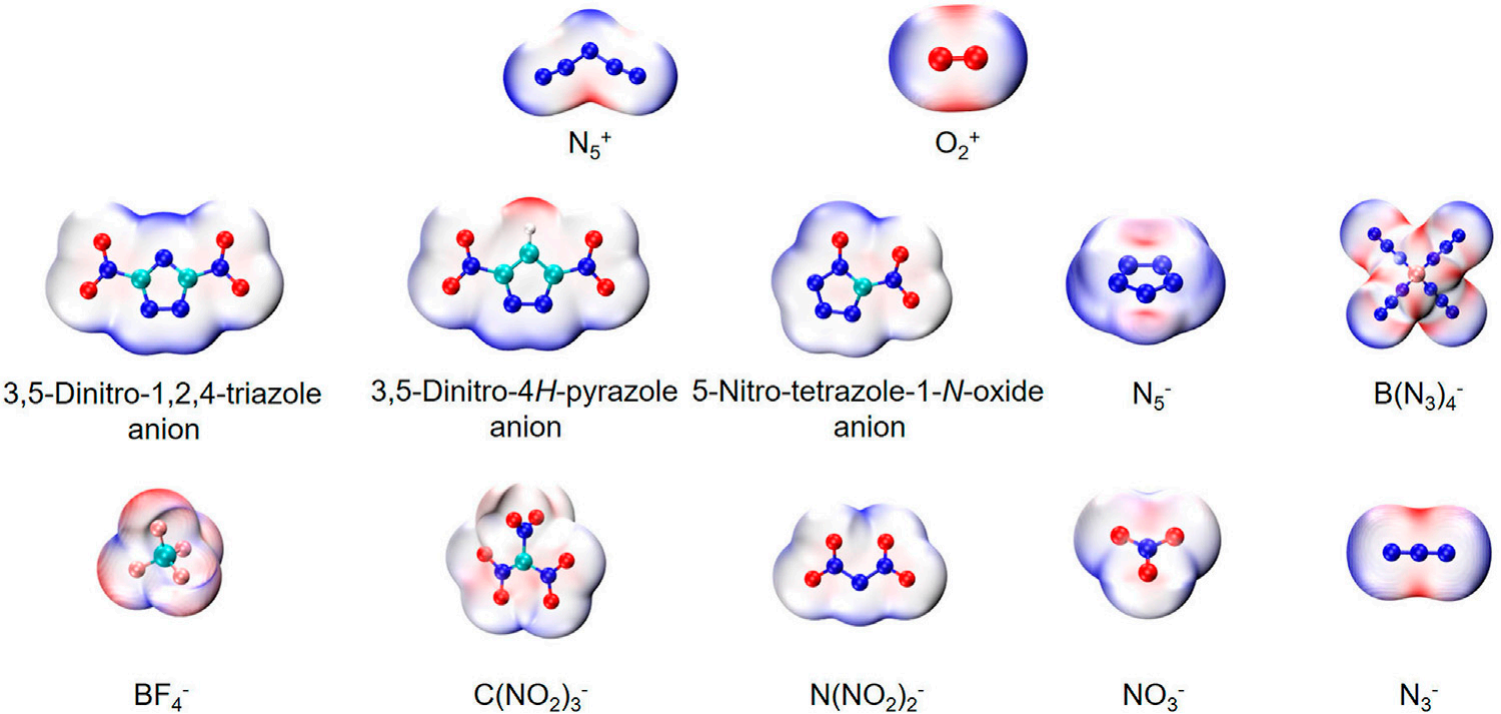
The molecular van der Waals surface electrostatic potential distribution of anions and cations. The red region represents the higher electrostatic potential in the molecular and blue region represents the lower electrostatic potential in it.

Figure 1 shows two types of energetic ionic salt compounds in this paper. For N_5_^+^ salts (A to J), all the anions are located inside the V-shape of the N_5_^+^. These spatial relative configurations are consistent with previous studies (Wang et al., 2011; Lian et al., 2012; Yu et al., 2015). The reason for this spatial distribution may be due to the electrostatic potential distribution on the surface of the N_5_^+^. Similarly, the structures of the O_2_^+^ salts are A1 to J1. The intrinsic cause of this spatial structure distribution remains the distribution of electrostatic potential on the surface of anions and cations. As can be seen from Figure 2, the high electrostatic potential portions of N_5_^+^ and O_2_^+^ are mainly concentrated in the V-shaped inner region of the N_5_^+^ (red region) and in the direction vertical to the bond axis of the O-O bond in O_2_^+^ (red region). This high positive electrostatic potential region tends to interact with the low negative electrostatic potential in the anion, thus affecting the molecular conformations of the ionic salts in Figure 1.

### 3.2 Crystal density

Crystal density is a critical consideration for energetic materials. In this section, the effects of different anions on the density of O_2_^+^ and N_5_^+^ salts are investigated separately. Table 1 lists the volumes, relevant parameters and densities of the individual ionic salt. It is easy to find that the densities of these two classes of ionic salts are designed to be between 1.490 and 2.263 g cm^−3^.

**TABLE 1:** The crystal densities (g cm^−3^) and volumes (cm^3^ mol^−1^) of N_5_^+^ (**A**-**J**) and O_2_^+^ salts (**A1**-**J1**).

Salts	*V*	$V_{s^{+}}$	$A_{s}^{+}$	$V_{s^{-}}$	$A_{s}^{-}$	ρ
**A**	221.181	128.687	91.277	−90.988	160.682	1.829
**B**	227.326	128.687	91.277	−89.434	164.537	1.775
**C**	195.731	128.687	91.277	−98.713	136.468	1.807
**D**	150.759	128.687	91.277	−118.677	94.990	1.626
**E**	265.753	128.687	91.277	−76.187	218.036	1.677
**F**	140.178	128.687	91.277	−124.560	90.170	1.944
**G**	206.867	128.687	91.277	−96.324	147.161	1.881
**H**	171.494	128.687	91.277	−107.599	117.227	1.806
**I**	134.838	128.687	91.277	−127.760	83.231	1.700
**J**	130.518	128.687	91.277	−129.171	79.263	1.490
**A1**	178.071	180.048	43.677	−90.988	160.682	2.030
**B1**	184.216	180.048	43.677	−89.434	164.537	1.961
**C1**	152.620	180.048	43.677	−98.713	136.468	2.014
**D1**	107.649	180.048	43.677	−107.599	117.227	2.028
**E1**	222.643	180.048	43.677	−76.187	218.036	1.834
**F1**	97.066	180.048	43.677	−124.560	90.170	2.263
**G1**	163.757	180.048	43.677	−96.324	147.161	2.102
**H1**	128.384	180.048	43.677	−107.599	117.227	2.028
**I1**	91.728	180.048	43.677	−127.760	83.231	1.917
**J1**	87.408	180.048	43.677	−129.171	79.263	1.610

On the whole, the density of salts formed by anion and O_2_^+^ is higher than that of ionic salts formed by anion and N_5_^+^. Compared with O_2_^+^, the volume of N_5_^+^ with V-shaped structure is larger than that of O_2_^+^, which is not conducive to the formation of dense accumulation, resulting in the density of this kind of ionic salts are generally lower than these of O_2_^+^. Among all ionic salts, A, C, F, G, H for N_5_^+^ salts and A1 to I1 for O_2_^+^ salts with a density higher than 1.800 g cm^−3^, which indicate that these compounds maybe have high detonation velocity. The densities of most O_2_^+^ salts are higher than those of HMX (1.90 g cm^−3^) (Wang et al., 2018) and RDX (1.80 g cm^−3^) (Wang et al., 2018), while the densities of A1, C1, D1, F1, G1 and H1 are close to that of CL-20 (2.04 g cm^−3^) (Fischer et al., 2015). In addition, the densities of H and I calculated in literature are 1.88 and 1.81 g cm^−3^, respectively, which higher than those are predicted in this paper (1.806 g cm^−3^ for H and 1.700 g cm^−3^ for I). This difference may be caused by the different calculation methods and the fact that the corrected volume is not used in this paper.

**FIGURE 3:**
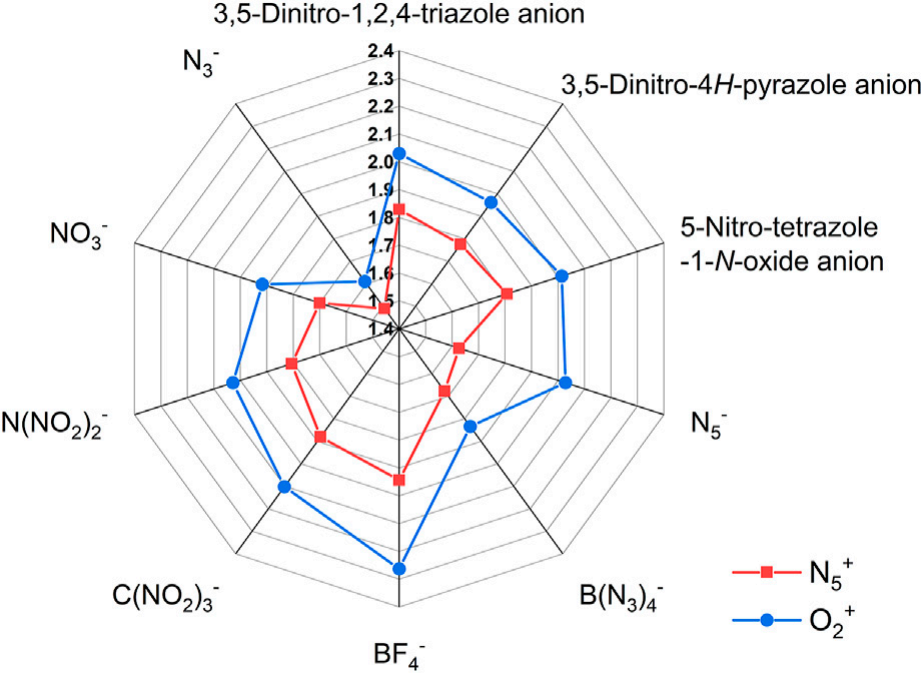
The radar plot of density of ionic salts containing O_2_^+^ and N_5_^+^.

In addition, the effect of different anions on the density of these two types of ionic salts is reflected in Figure 3, respectively. In Figure 3, the blue closed line wraps the red one, indicating that the density of the O_2_^+^ salts is greater than that of the N_5_^+^ salts. Among all ionic salts, the salts formed by BF_4_^−^ anion have the highest density, reaching 1.944 g cm^−3^ (1.99 g cm^−3^ in the literature (Wang et al., 2011)) for N_5_^+^ salts and 2.263 g cm^−3^ for O_2_^+^ salts. However, salts formed from N_3_^−^ have the lowest densities, 1.490 g cm^−3^ for N_5_^+^ salts and 1.610 g cm^−3^ for O_2_^+^ salts, respectively. The radar plot shows that the order of the effect of different anions on the ionic salts density are BF_4_^−^ > C(NO_2_)_3_^-^ > 3,5-Dinitro-1,2,4-triazole anion > 5-Nitro-tetrazole-1-*N*-oxide anion > N(NO_2_)_2_^-^ > 3,5-Dinitro-4*H*-pyrazole anion > NO_3_^−^ > B(N_3_)_4_^-^ > N_5_^−^ > N_3_^−^ for N_5_^+^ salts and BF_4_^−^ > C(NO_2_)_3_^-^ > 3,5-Dinitro-1,2,4-triazole anion > N_5_^−^ = N(NO_2_)_2_^-^ > 5-Nitro-tetrazole-1-*N*-oxide anion >3,5-Dinitro-4*H*-pyrazole anion > NO_3_^−^ > B(N_3_)_4_^-^ > N_3_^−^ for O_2_^+^ salt.

### 3.3 Enthalpy of formation

A high and positive enthalpy of formation is a typical feature of energetic compounds. In this section we study the enthalpy of formation of the designed ionic salts. Table 2 contains the formation enthalpies of each anion ( $∆H_{f}\left(anion\right)$ ), cation ( $∆H_{f}\left(cation\right)$ ) and corresponding salt ( $∆H_{f}$ ), and the lattice energy ( $∆H_{L}$ ) of each salt.

**TABLE 2:** The enthalpy of formation of O_2_^+^ and N_5_^+^, various anions and their corresponding salts (∆*H*_*f*_) (**A** to **J** for N_5_^+^ salts and **A1** to **J1** for O_2_^+^ salts**)**, as well as lattice energies (∆*H*_*L*_) of these salts. (unit, kJ/mol).

Salts	${∆H}_{f}^{a}\left(cation\right)$	${∆H}_{f}^{b}\left(anion\right)$	$∆H_{L}^{c}$	$∆H_{f}^{d}$
**A**	1496.733	106.228	505.305	1097.656
**B**	1496.733	83.104	501.934	1077.903
**C**	1496.733	238.726	521.344	1214.115
**D**	1496.733	316.461	557.376	1255.817
**E**	1496.733	849.433	482.932	1863.234
**F**	1496.733	−1724.827	567.272	−795.366
**G**	1496.733	−90.096	513.820	892.816
**H**	1496.733	−30.616	539.262	926.855
**I**	1496.733	−244.451	573.084	679.198
**J**	1496.733	226.004	578.134	1144.603
**A1**	1197.073	106.228	543.823	759.478
**B1**	1197.073	83.104	539.582	740.595
**C1**	1197.073	238.726	566.424	869.375
**D1**	1197.073	316.461	623.040	890.494
**E1**	1197.073	849.433	514.958	1531.548
**F1**	1197.073	−1724.827	636.601	−1164.355
**G1**	1197.073	−90.096	555.300	551.677
**H1**	1197.073	−30.616	592.801	573.656
**I1**	1197.073	−244.451	648.782	303.840
**J1**	1197.073	226.004	660.504	762.573

a Gaseous enthalpy of formation of cations.

b Gaseous enthalpy of formation of anions.

c Lattice energy.

d Enthalpy of formation of ionic salts.

It is easy to find from Table 2 that the enthalpy of formation of salts formed by anion and N_5_^+^ is higher than that of ionic salt formed by anion and O_2_^+^. The O_2_^+^ and N_5_^+^ have very high enthalpy of formation (both >1000 kJ/mol), which maybe determine the ultra-high enthalpy of formation for these ionic salts. As deduced earlier, the enthalpy of formation of all the remaining ionic salts are positive except for F and F1. Among them, the highest enthalpy of formation is E (up to 1863.23 kJ/mol) and the lowest is I1 (up to 303.84 kJ/mol). Except for F, the enthalpy of formation of the remaining N_5_^+^ salts are all much higher than that of CL-20 (365.4 kJ/mol) (Wang et al., 2018). Although the enthalpy of formation of O_2_^+^ salts is not as high as that of N_5_^+^ salts, most of the salts also have a fairly high enthalpy of formation (1531.55 kJ/mol for **E1**).

**FIGURE 4:**
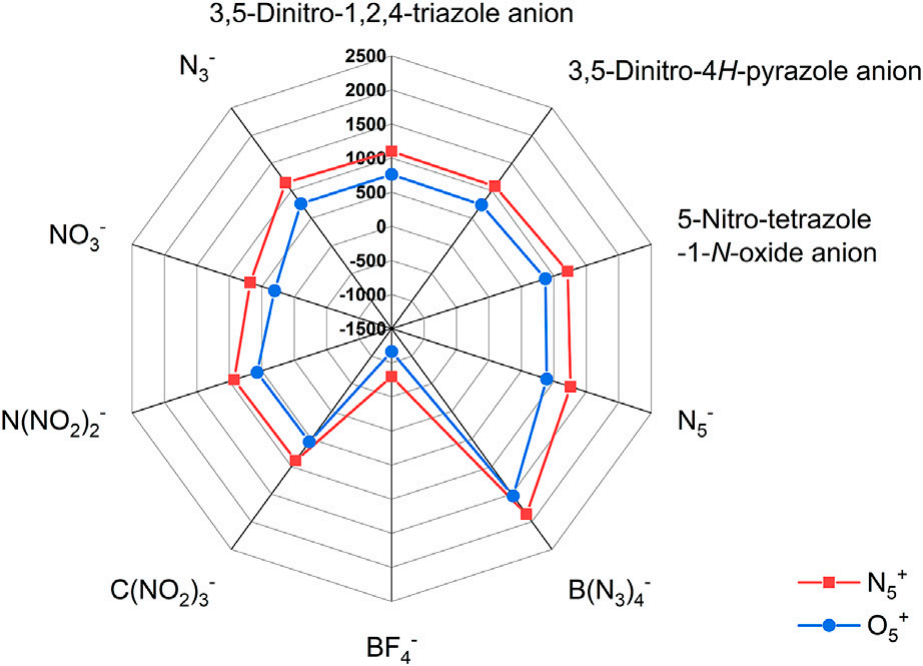
The radar plot of enthalpy of formation of ionic salts containing O_2_^+^ and N_5_^+^.

In addition, the effect of different anions on the enthalpy of formation of these two types of ionic salts is reflected in Figure 4, respectively. In Figure 4, the red closed line wraps the blue one, indicating that the enthalpy of formation of the N_5_^+^ salts is greater than that of the O_2_^+^ salts. Among all ionic salts, the salts formed by B(N_3_)_4_^-^ have the highest enthalpy of formation, reaching 1863.23 kJ/mol for N_5_^+^ salts and 1531.55 kJ/mol for O_2_^+^ salts, which implies that the B(N_3_)_4_^-^ can significantly increase the enthalpy of formation of the compound. However, salts formed from BF_4_^−^ anions have the lowest enthalpy of formation, −795.37 kJ/mol for N_5_^+^ salts and −1164.36 kJ/mol for O_2_^+^ salts, respectively. The introduction of O_2_^+^ and N_5_^+^ with ultra-high enthalpy of formation in compounds is an effective way to increase the enthalpy of formation of compounds. However, fluorine sharply reduces the enthalpy of formation of the compound, so it causes F (−795.37 kJ/mol) and F1 (−1164.355 kJ/mol) to have ultra-low enthalpies of formation. The radar plot shows that the order of the effect of different anions on enthalpy of formation of the ionic salts is B(N_3_)_4_^-^ > N_5_^−^ > 5-Nitro-tetrazole-1-*N*-oxide anion > N_3_^−^ > 3,5-Dinitro-1,2,4-triazole anion > 3,5-Dinitro-4*H*-pyrazole anion > N(NO_2_)_2_^-^ > C(NO_2_)_3_^-^ > NO_3_^−^ > BF_4_^−^, regardless of whether it is an O_2_^+^ salt or an N_5_^+^ salt.

### 3.4 Energetic properties

The explosive properties of energetic materials are to evaluate the energy performance of the characteristic parameters, mainly including the detonation velocity (*D*), detonation pressure (*P*), detonation heat (Q), etc. Table 3 contains the *D*, *P*, *Q* and specific impulse (*I*_sp_) for all designed ionic salts.

**TABLE 3:** Predicted explosive properties, specific impulse (Isp) and oxygen balance of N_5_^+^ and O_2_^+^ salts.

Salts	*Q* ^a^ (kJ kg^−1^)	*D* ^b^ (km s^−1^)	*P* ^c^ (GPa)	*I*_sp_ ^d^ (s)	OB^e^ (%)
**A**	7797.40	9694	40.58	295.46	0
**B**	7628.79	9592	40.78	305.06	−17.61
**C**	7793.69	9765	40.59	303.91	8.00
**D**	9026.90	9778	38.16	348.23	0
**E**	8560.77	9331	35.06	305.98	−9.64
**F**	2487.00	7,162	20.15	166.54	5.10
**G**	5830.04	9128	35.67	270.07	29.08
**H**	5247.90	8920/8859^f^	32.76/32.3^f^	268.18	63.65
**I**	5103.58	8433/8642^f^	28.02/30.3^f^	265.72	63.65
**J**	10,196.80	9402	33.83	367.67	0
**A1**	8086.01	9985	46.71	286.96	16.84
**B1**	9579.26	9983	47.73	302.66	−4.23
**C1**	7701.67	10,025	46.92	293.26	29.61
**D1**	8390.83	9895	42.69	324.52	31.36
**E1**	8686.17	10,833	56.63	349.70	3.79
**F1**	—	—	—	—	—
**G1**	5219.46	9226	39.53	254.96	52.73
**H1**	4198.91	8737	33.80	241.48	69.55
**I1**	3288.32	7605	24.27	216.79	85.10
**J1**	9389.90	9174	34.95	340.44	43.23

a Explosive heat.

b Detonation velocity.

c Explosive pressure.

d Specific impulse.

e Oxygen balance.

f Detonation velocity and detonation pressure calculated in literature (Sun et al., 2020).

It is easy to find that most of the ionic salts have *D* in excess of 8,000 m/s. Among the N_5_^+^ salts, all except F (7,162 m/s), H (8,920 m/s) and I (8,433 m/s) have *D* exceeding 9000 m/s, owing to their high density. Similarly, among the O_2_^+^ salts, the *D* exceeded 9000 m/s for all salts except H1 (8,737 m/s), I1 (7,605 m/s) and F1. Excitingly, the *D* of C1 (10,025 m/s) and E1 (10,833 m/s) exceeded 10,000 m/s, and the *D* of A1 (9985 m/s), B1 (9983 m/s) and D1 (9895 m/s) were close to 10,000 m/s, which fully demonstrates the potential of O_2_^+^ as active ingredients of ultra-high energy oxidants. The introduction of O_2_^+^ into the system significantly increases the crystal density and oxygen balance, which fundamentally determines the high detonation velocity properties of this category of salts.

The *P* is another important index of energetic materials. Compared with the *P* of CL-20, the *P* of A1, B1, C1, and E1 are all higher than that of CL-20 in O_2_^+^ salts, reaching 46.71, 47.73, 46.92, 56.63 GPa, respectively. In the case of N_5_^+^ salts, although the highest *p* value is not as high as E1 in O_2_^+^ salts, the *P* of some N_5_^+^ salts (A, B, C) also reaches more than 40 GPa.

Oxygen balance is an important reference indicator for screening energetic materials. As can be seen in Table 3, the oxygen balance of the O_2_^+^ salts are higher than these of the N_5_^+^ salts for salts composed of the same anion, which directly indicates that the way to improve the oxygen balance is to introduce O_2_^+^ into the system. Energetic ionic salts can be used not only as energetic materials, but also as propellants. The value of *I*_sp_ can be used to compare the performance of different rocket propellants. Among the N_5_^+^ salts, the *I*_sp_ is higher than 260 s for all ionic salts except F (166.54 s). Five N_5_^+^ salts have *I*_sp_ value exceeding 300 s, with the highest value of J reaching 367.67 s. In contrast, among the O_2_^+^ salts, there are four ionic salts with *I*_sp_ exceeding 300 s, namely B1 (302.66 s), D1 (324.52 s), E1 (349.70 s), and J1 (340.44 s). The ultra-high *I*_sp_ value further illustrate the promise of these super-oxidizing ionic salts to drive further development of conventional propellants.

**FIGURE 5:**
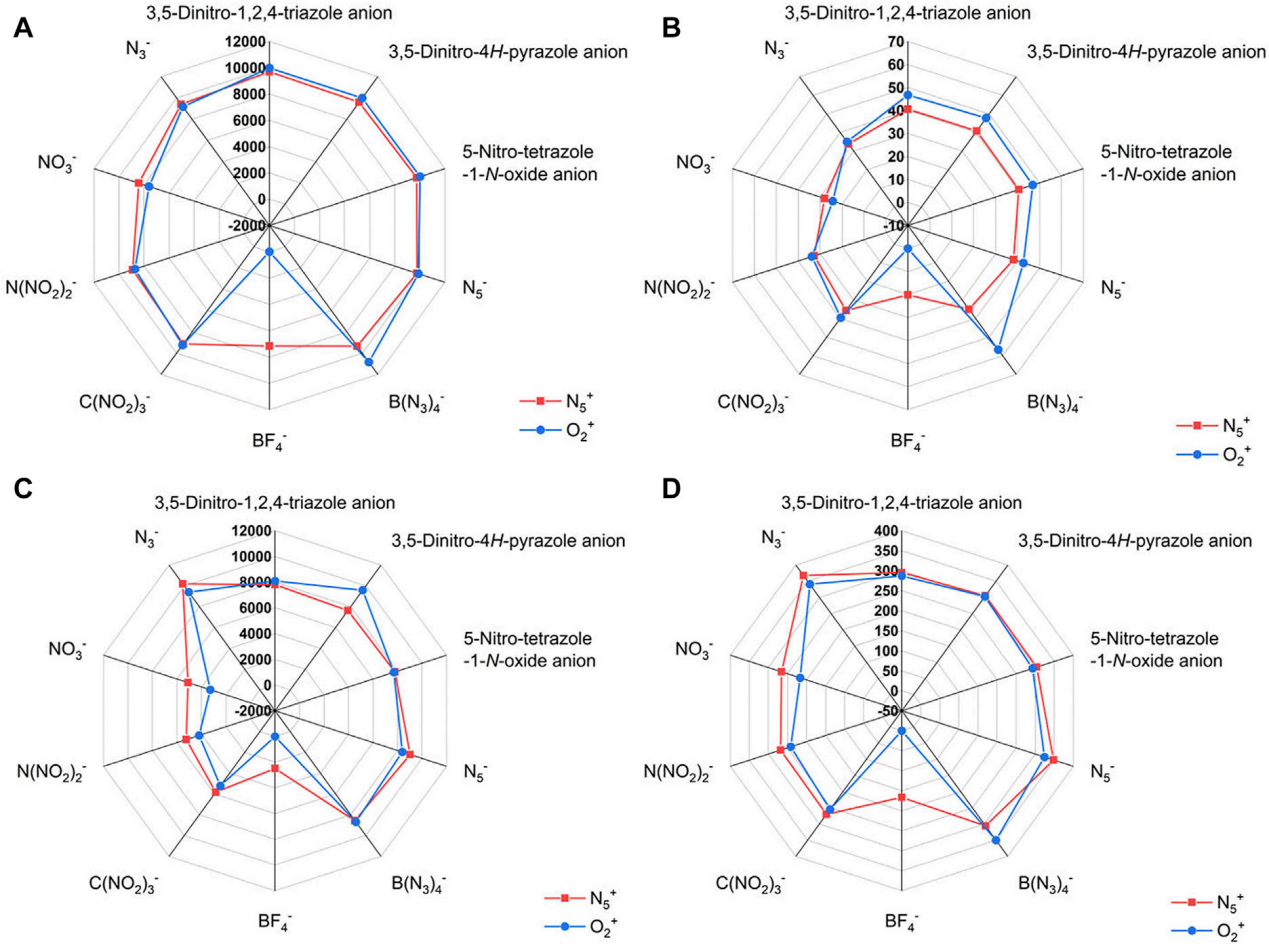
The radar plots of the effect of different anions on the detonation parameters of the N_5_^+^ and O_2_^+^ salts. The **(A)** is for *D*, **(B)** is for *P*, **(C)** is for *Q* and **(D)** is for *I*_sp_, respectively.

In addition, Figure 5 shows the effect of different anions on the detonation velocity A, detonation pressure B, detonation heat C and specific impulse D. Based on the degree of overlap of the two curves (the blue and the red) in the radar plots, we can clearly find the effect of different anions on the detonation parameters of the ionic salts.

Among these ten anion structures, it is not possible to calculate the detonation parameters of the O_2_^+^ salts composed of BF_4_^−^ by the Kamlet−Jacobs formula. The reason for this phenomenon may be due to the ultra-low enthalpy of formation of this ionic salts (−1164.355 kJ/mol for F1). As a comparison, the ionic salt formed by BF_4_^−^ and N_5_^+^ has a calculated detonation parameter, which indicates that the N_5_^+^ enhances the energy of this system more than the O_2_^+^ (enthalpy of formation of F is −795.37 kJ/mol). It is obvious from the radar plot (Figures 5C,D) that the N_3_^−^ has a greater influence on the heat of explosion and specific impulse than other anions. The effects of 3,5-Dinitro-1,2,4-triazole anion, 3,5-Dinitro-4*H*-pyrazole anion, 5-Nitro-tetrazole-1-*N*-oxide anion and N_5_^−^ on the detonation velocity are similar in both N_5_^+^ and O_2_^+^ salts (Figure 5A). B(N_3_)_4_^-^ has great influence on the detonation velocity, detonation pressure and specific impulse of O_2_^+^ salt (Figures 5A,B,D). Among the N_5_^+^ salts, the detonation performance of the ionic salt formed by the BF_4_^−^ is lower than that of the other anions (Figures 5A–D). This illustrates that the element fluorine is not conducive to the detonation performance of a single-compound energetic material. In ionic salts consisting of C(NO_2_)_3_^-^, N(NO_2_)_2_^-^ and NO_3_^−^, the detonation properties of the ionic salts gradually increase with the increase in the number of NO_2_ groups.

### 3.5 Impact sensitivity

Impact sensitivity (H_50_) is used to describe the degree of difficulty of explosion of energetic materials under impact stimulation, which indicates the stability of compounds to a certain extent. The higher the value of H_50_, the more stable the compound is to shock stimulation. Table 4 lists the positive variance ( $\sigma_{+}^{2}$ ), balance of charges (*υ*) and H_50_ of each ionic salt. The computational results show that the H_50_ of these ionic salts are between 13.27 cm (I1) and 54.63 cm (I). Since the sensitivity of energetic materials is affected by many factors, the impact sensitivity here can only be used as a reference. Forcibly analyzing this part of the content is very likely to get meaningless or even wrong conclusions.

**TABLE 4:** Values of H_50_ for N_5_^+^ and O_2_^+^ salts.

Salts	$\sigma_{+}^{2}$ (kcal/mol)^2^	*υ*	H_50_ (cm)
A	147.31	0.1599	34.23
B	109.04	0.1875	41.15
C	152.62	0.1856	40.40
D	225.06	0.2464	54.61
E	202.13	0.1528	32.17
F	239.91	0.2222	48.69
G	222.65	0.1896	40.93
H	202.54	0.1926	41.77
I	141.78	0.2443	54.63
J	97.87	0.2193	48.89
A1	96.35	0.0929	18.37
B1	144.31	0.1239	25.55
C1	86.65	0.1415	30.17
D1	70.77	0.2277	51.10
E1	60.76	0.2268	50.93
F1	88.54	0.1619	35.10
G1	69.70	0.0997	20.18
H1	67.51	0.0792	15.26
I1	58.23	0.0707	13.27
J1	60.25	0.1221	25.66
N_5_AsF_6_	288.82	0.1664	34.91
N_5_SbF_6_	294.90	0.1559	32.32

### 3.6 Stability

The newly designed high-energy oxidizing ionic salts need to have not only excellent detonation performance, but also sufficient stability. As an allotrope of N_5_^+^, the N_5_^−^ was successfully synthesized by researchers in 2017 (Xu et al., 2017; Zhang et al., 2017). It is believed that the N_5_^−^ is stable at atmospheric pressure because of its ability to form coordination interactions with metal cations and to form hydrogen bonds with water molecules. Subsequently, a series of ionic salts formed by N_5_^−^ and organic nitrogen-rich cations (Yang et al., 2018; Xu et al., 2019) were designed and synthesized, which further broadened the study of N_5_^−^ ions.

**FIGURE 6:**
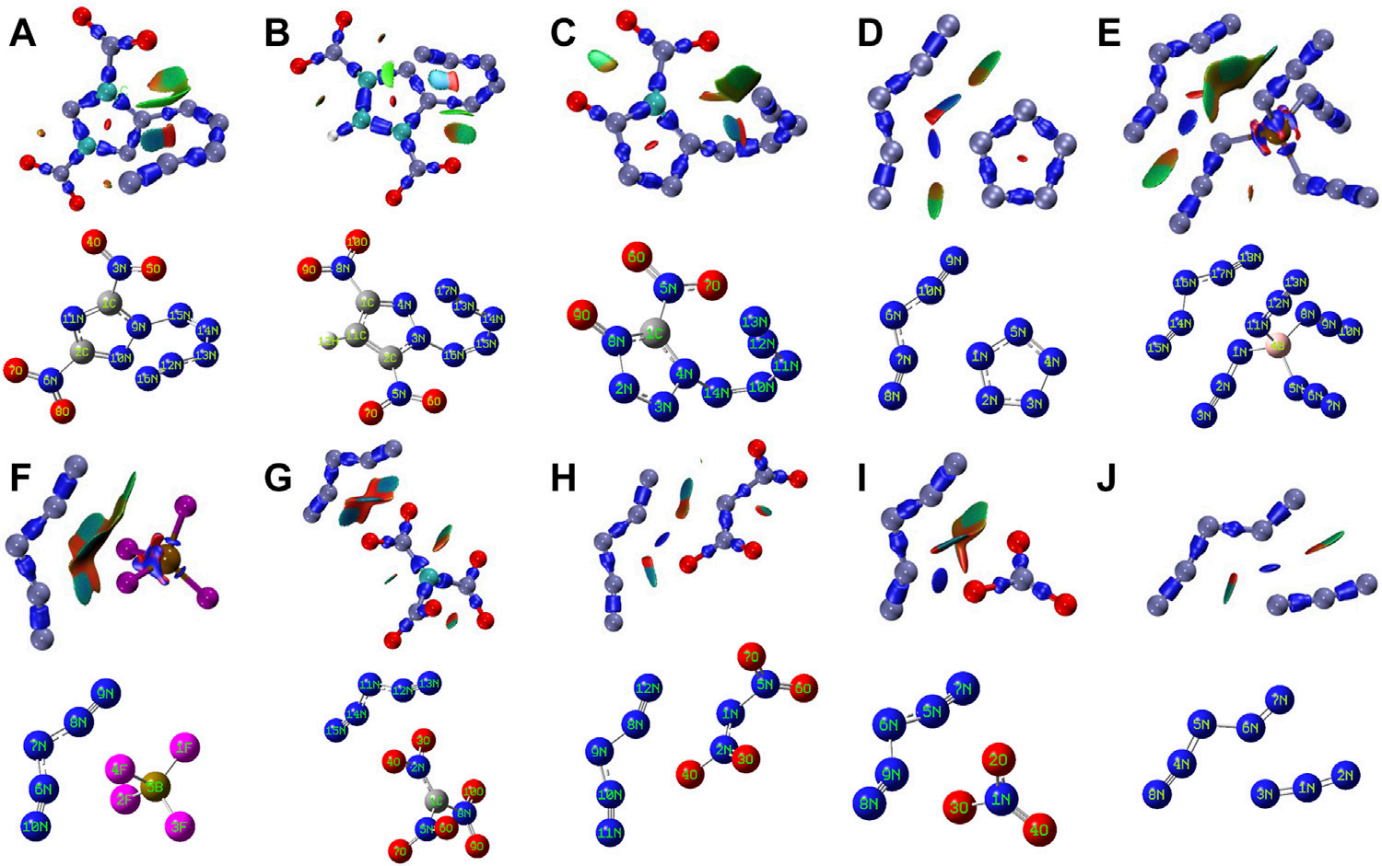
Isosurface map of IRI and molecular structures of N_5_^+^ salts in this study.

Based on this stabilization mechanism, we hypothesize that the N_5_^+^ is also capable of stabilizing with certain nonmetallic anions through electrostatic interactions or other van der Waals interactions. Therefore, we first analyzed the various interactions (see Figure 6) existing within the ionic salts by interaction region indicator (IRI) (Lu and Chen, 2021) analyses, and then calculated the Gibbs free energies change of formation of the ionic salts as ways of which to illustrate the stability of the designed energetic ionic salts.

In Figure 6, for N_5_^+^ salts, the presence of dark blue portions of N9-N15 in **A**, N3-N6 in B and N4-N14 in **C**, respectively, can be found in the IRI isosurface map, implying that all these bonds are relatively strong covalent chemical bonds. In D, E, and H, the presence of dark blue regions at the space outside the ionic fragment, which implies the presence of strong interactions in these parts. However, there is no dark blue region in F, G and J, which is similar to that in D, E and H, suggesting that the binding degree of cation and anion in these three compounds is relatively weak. However, for O_2_^+^ salts in Supplementary Figure S1), except D1, there is no obvious covalent interaction between anions and cations in other salts. The results show that in F1, BF_4_^−^ seems to dissociate, forming F^−^ and BF_3_ groups, and there is a strong interaction between O_2_^+^ and F^−^ in F1. In other ionic compounds, O_2_^+^ seems to form a strong van der Waals interaction with anions (green IRI isosurface rather than dark blue isosurface), and a strong interaction (blue IRI isosurface) appears to be formed between O_2_^+^ and N_5_^+^ in D1. In short, multiple interactions within ionic compounds contribute to the stability of their own structures.

**FIGURE 7:**
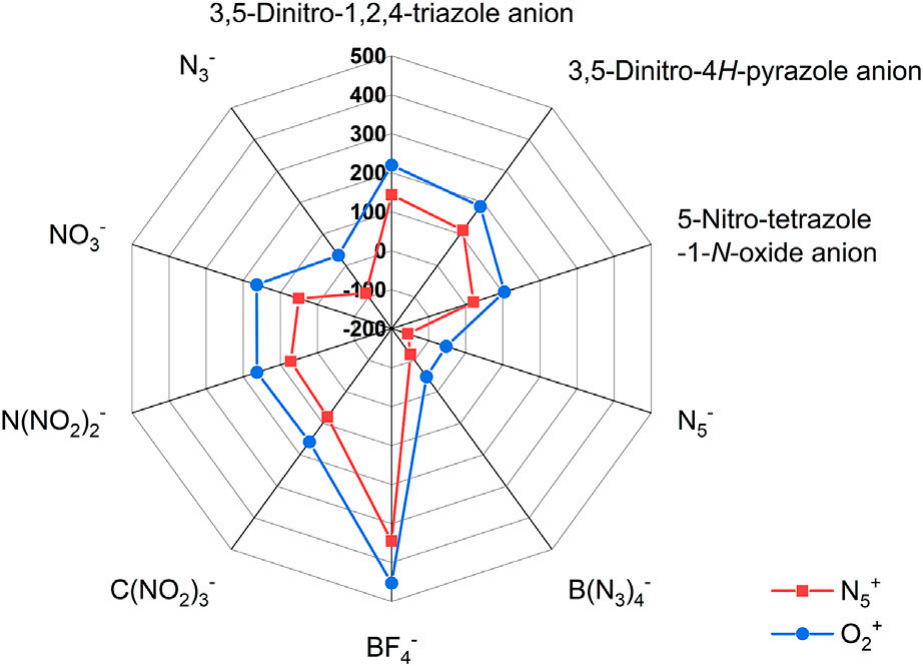
The radar plots of the effect of different anions on the Gibbs free energy change of the formation of the N_5_^+^ and O_2_^+^ salts.

In addition, Table 5 lists the Gibbs free energy change of the formation of each ionic salt. It is easy to find that except for a few ionic salts (D, E, and J in N_5_^+^ salts and D1, E1 in O_2_^+^ salts), the Gibbs free energy changes of the formation of other ionic salts are positive, indicating that these ionic salt structures can exist stably in thermodynamics. Figure 7 represents the effect of different anions on the Gibbs free energy of ionic salt generation. Relative to the other anions, the BF_4_^−^ has the highest Gibbs free energy change of the formation of each ionic salt (345.36 kJ/mol for F and 452.44 kJ/mol for F1). In contrast, ionic salts containing N_5_^−^ have the lowest Gibbs free energy change of the formation (-156.27 kJ/mol for D and-52.861 kJ/mol for D1). The negative Gibbs free energy change of the formation indicate that both D and D1 cannot exist stably, which is consistent with the conclusions of Christe’s study (Dixon et al., 2004). Also, our findings indicate that the free energy change of the ionic salt J formed from the N_3_^−^ and N_5_^+^ is also negative, implying that this ionic salt also cannot exist stably, which is once again consistent with the conclusion of Christe’s study. However, the Gibbs free energy change of the formation of J1, an ionic salt formed from N_3_^−^ and O_2_^+^, becomes positive, implying that this compound can be thermodynamically stable. Finally, the order of the influence of other anions on the Gibbs free energy change of ionic salt are: BF_4_^−^ > 3,5-Dinitro-1,2,4-triazole anion >3,5-Dinitro-4*H*-pyrazole anion > C(NO_2_)_3_^-^ > N(NO_2_)_2_^-^ > NO_3_^−^ > 5-Nitro-tetrazole-1-*N*-oxide anion > N_3_^−^ > B(N_3_)_4_^-^ > N_5_^−^ for N_5_^+^ ionic salts or BF_4_^−^ > 3,5-Dinitro-1,2,4-triazole anion >3,5-Dinitro-4*H*-pyrazole anion > NO_3_^−^ > N(NO_2_)_2_^-^ > C(NO_2_)_3_^-^ > 5-Nitro-tetrazole-1-*N*-oxide anion > N_3_^−^ > B(N_3_)_4_^-^ > N_5_^−^ for O_2_^+^ ionic salts.

**TABLE 5:** Calculated adiabatic ionization potential (*AIP*), adiabatic electron affinity (*AEA*), lattice energy ( $∆H_{L}$ ), reaction enthalpy change ( $∆H_{rxn}$ ), entropy change ( $∆s_{rxn}$ ) and Gibbs free energy change of formation ( $∆G_{rxn}$ ) for N_5_^+^ salts and O_2_^+^ salts.

Salts	*AIP* ^a^	*AEA* ^b^	$∆H_{L}$ ^c^	$∆H_{rxn}$ ^d^	$∆s_{rxn}$ ^e^	$∆G_{rxn}$ ^f^
A	525.925	−1017.967	505.305	13.262	−435.673	143.093
B	500.468	−1017.967	501.934	−15.566	−427.389	111.796
C	394.0858	−1017.967	521.344	−102.539	−414.667	21.032
D	200.414	−1017.967	557.376	−260.177	−348.683	−156.270
E	310.204	−1017.967	482.932	−224.832	−360.604	−117.372
F	682.290	−1017.967	567.272	231.514	−382.040	345.362
G	448.287	−1017.967	513.820	−55.860	−455.085	79.756
H	429.914	−1017.967	539.262	−48.791	−405.680	72.101
I	387.624	−1017.967	573.084	−57.259	−361.632	50.507
J	253.111	−1017.967	578.134	−186.721	−332.297	−87.697
A1	525.925	−966.911	543.823	102.837	−391.005	219.356
B1	500.468	−966.911	539.582	73.139	−382.720	187.189
C1	394.085	−966.911	566.424	−6.403	−369.998	103.856
D1	200.414	−966.911	623.040	−143.457	−304.015	−52.861
E1	310.204	−966.911	514.958	−141.749	−315.936	−47.600
F1	682.209	−966.911	636.601	351.899	−337.371	452.435
G1	448.287	−966.911	555.300	36.676	−410.416	158.980
H1	429.914	−966.911	592.801	55.804	−361.012	163.385
I1	387.624	−966.911	648.782	69.495	−316.964	163.950
J1	253.111	−966.911	660.504	−53.296	−287.627	32.418

a Adiabatic ionization potential.

b Adiabatic electron affinity.

c Lattice energy.

d Enthalpy change of reaction.

e Entropy change of reaction.

f Gibbs free energy change of formation.

## 4 Conclusion

The combination of high-energy oxidants N_5_^+^ and O_2_^+^ with high nitrogen organic anions is a new design idea proposed with the help of quantum chemical calculations. This idea can not only ensure the overall high energy level of ionic salt, but also consider the thermodynamic stability. Most ionic salts have good detonation velocity and pressure because of their high density and enthalpy of formation, suggesting these ionic salts have potential as candidates for high-energy oxidants. The introduction of O_2_^+^ into the system is an important means to improve the ionic salt oxygen balance. Considering the energy properties (enthalpy of formation, detonation velocity, detonation pressure, detonation heat, specific impulse) and stability (formation of Gibbs free energy) of these designed ionic salts, we believe that A, B, C, G, H for N_5_^+^ salts and A1, B1, C1, G1, H1 for O_2_^+^ salts are expected to be further studied and experimentally developed.

## Data Availability

The raw data supporting the conclusion of this article will be made available by the authors, without undue reservation.
