# Supplementary figures and images for: The ionic salts with super oxidizing ions O2 + and N5 +: Potential candidates for high-energy oxidants
Source: Front Chem. 2022 Sep 21;10:1005816. doi: 10.3389/fchem.2022.1005816 (PMC9532705; doi:10.3389/fchem.2022.1005816)

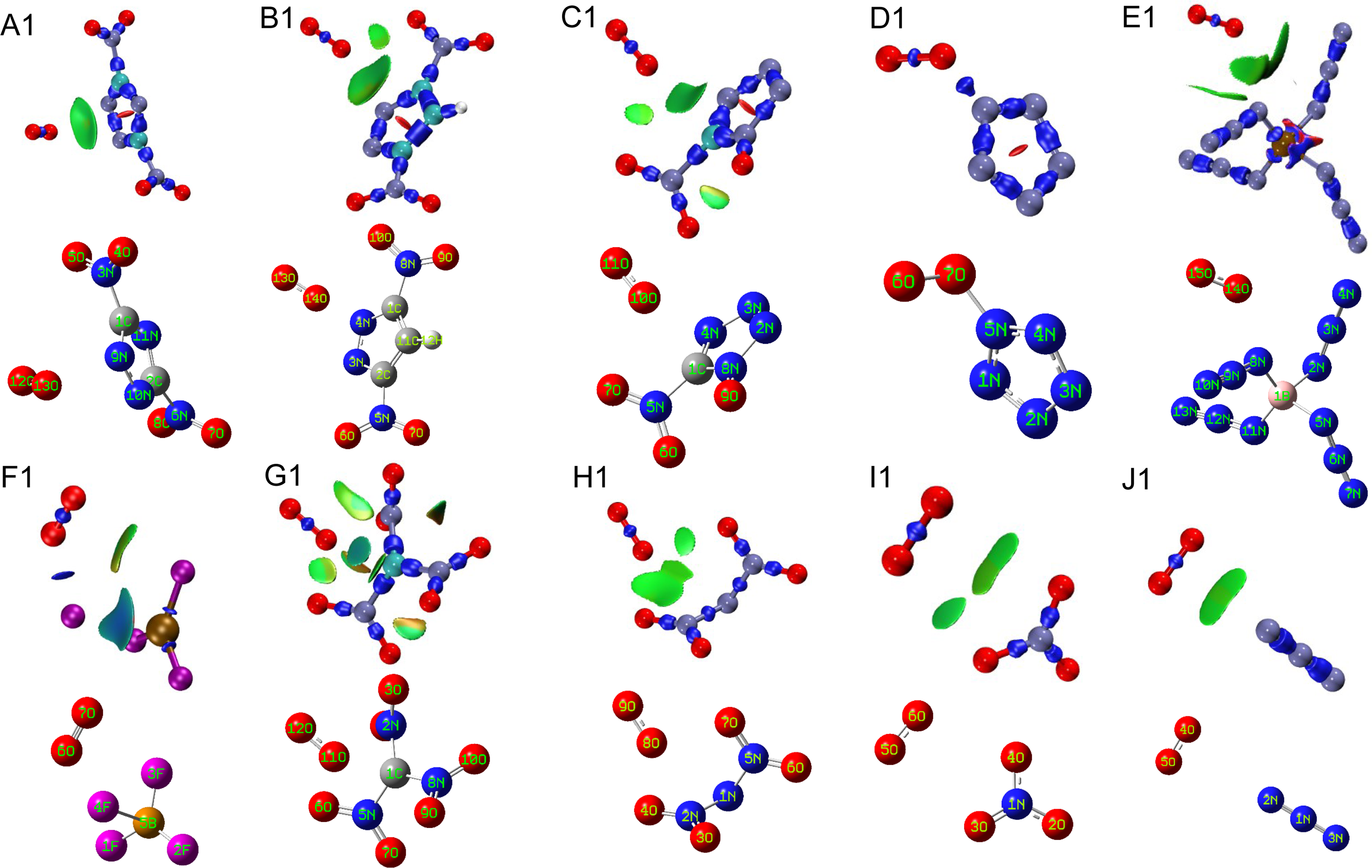

Supplement: Supplementary file 1 [file Image1.TIF]
